# Curación de úlcera venosa crónica de la pierna con aloinjerto de membrana amniocoriónica humana fresca

**DOI:** 10.7705/biomedica.6319

**Published:** 2022-05-01

**Authors:** Alberto Piamo, Mayra García, Dayset Romero, Daisy Ferrer

**Affiliations:** 1 Servicio de Anatomía Patológica, Hospital Maternoinfantil de Amazonas, Puerto Ayacucho, Venezuela Hospital Maternoinfantil de Amazonas Puerto Ayacucho Venezuela; 2 Servicio de Gineco-obstetricia, Hospital Maternoinfantil de Amazonas, Puerto Ayacucho, Venezuela Hospital Maternoinfantil de Amazonas Puerto Ayacucho Venezuela; 3 Servicio de Enfermería, Ambulatorio “Jacinto Convit” San Antonio de Cúa, Venezuela Ambulatorio “Jacinto Convit” San Antonio de Cúa Venezuela; 4 Facultad de Ciencias Médicas “ICBP Victoria de Girón”Universidad de Ciencias Médicas, La Habana, Cuba Universidad de Ciencias Médicas La Habana Cuba

**Keywords:** úlcera varicosa, aloinjertos, amnios., Varicose ulcer, allografts, amnion.

## Abstract

En su estado fresco, la membrana amniocoriónica contiene varias células multipotenciales, factores de crecimiento y proteínas de la matriz extracelular que contribuyen a la cicatrización de las úlceras vasculares crónicas. Para demostrar su efectividad, se recurrió a un aloinjerto de membrana placentaria humana fresca para tratar una úlcera venosa crónica, de 12 x 10 cm y con 40 años de evolución, en el zona maleolar interna e izquierda de una paciente de 89 años de edad. Transcurridos 60 días del injerto, la úlcera se encontraba cicatrizada en el 100 % de su superficie, observándose una cicatriz rosada clara en cuyos bordes se apreciaron intentos de pigmentación. El aloinjerto de membrana amniocoriónica humana fresca es una alternativa terapéutica para la curación de úlceras vasculares crónicas persistentes en las extremidades inferiores.

## Abstrac

In its fresh state, the amniochorionic membrane contains various multipotential cells, growth factors, and extracellular matrix proteins that contribute to the healing of chronic vascular ulcers. To demonstrate its effectiveness, a fresh human placental membrane allograft was applied to a chronic venous ulcer in the lower limb of an 89-year-old female patient with a 12 x 10 cm ulcerated lesion of 40 years of evolution in the malleolar area of her left lower limb. Sixty days after the graft, the ulcer was healed in 100% of its surface and a light pink scar on the edges indicated possible pigmentation. Fresh human amniochorionic membrane allograft is a therapeutic alternative for the healing of refractory chronic vascular ulcers of the lower extremities.

Las úlceras venosas crónicas en las piernas son lesiones abiertas de la extremidad inferior ^1^, localizadas entre la rodilla y la articulación del tobillo que permanecen sin cicatrizar durante al menos 30 días ^2^ y son causadas por insuficiencia venosa crónica ^3^^,^^4^. Estas lesiones pueden tardar meses o años en sanar y son propensas a la recurrencia ^5^, por lo que se convierten en una carga económica notable para los pacientes y los sistemas sanitarios ^6^.

Las intervenciones complementarias ampliamente utilizadas en la curación de este tipo de úlceras incluyen apósitos para heridas con componentes activos (los llamados apósitos avanzados para heridas), antimicrobianos locales o sistémicos y cirugía venosa ^7^.

Considerando que las guías de la *Wound Healing Society* recomiendan considerar terapias avanzadas para heridas si la úlcera no disminuye de tamaño en el 40 % o más después de cuatro semanas de terapia estándar ^8^, y que la *Society for Vascular Surgery, la American Podiatric Medical Association y la Society for Vascular Medicine* recomiendan la terapia adyuvante si el área de la herida no se reduce en más del 50 % después de un mínimo de cuatro semanas de tratamiento estándar ^9^, se decidió ofrecer un aloinjerto de membrana amniocoriónica humana fresca para tratar una úlcera venosa con 40 años de evolución en el miembro inferior izquierdo de una paciente de 89 años de edad, para evaluar su eficacia como tratamiento de cicatrización.

## Presentación de caso

**Figura 1:**
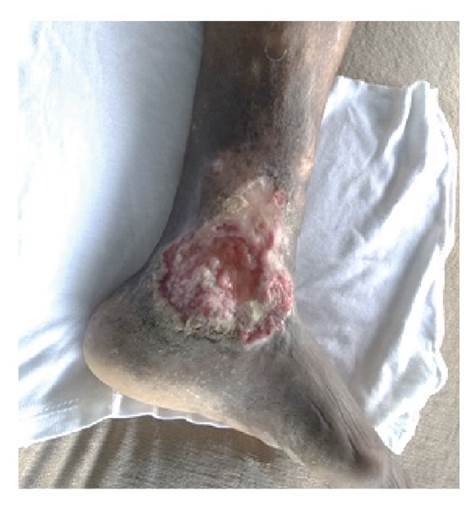
Lesión ulcerada de 40 años de evolución en región maleolar izquierda

Se trata de una paciente de 89 años de edad, de piel negra y antecedentes de demencia senil, que presentaba una lesión ulcerada de 12 x 10 cm y con 40 años de evolución en el área maleolar interna del miembro inferior izquierdo. La lesión era piriforme, con base inferior, bordes elevados y socavada en su centro en 2 mm, fondo hemorrágico y costroso alternado con zonas de exudado blanquecino adherido; hacia su periferia, se observaban áreas pálidas costrosas y, en la parte inferior, hiperqueratosis exuberante, en tanto que la piel circundante se encontraba hiperpigmentada (figura 1).

**Figura 2:**
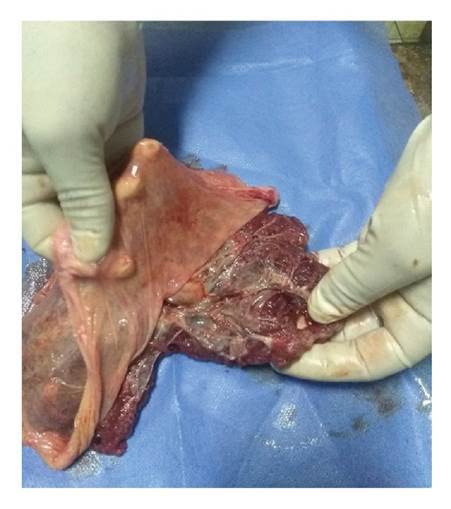
Proceso de disección de la membrana amniótica

Según refirieron los familiares, desde que apareció la úlcera la paciente había presentado múltiples complicaciones, entre las que destacaban la celulitis, la lipodermatoesclerosis, la miasis recurrente y la formación de microabscesos, por lo que requería frecuentemente múltiples ciclos de tratamientos antibióticos (amoxicilina, azitromicina, ciprofloxacino) y de medicina tradicional que habían producido ligeros intentos de cicatrización. La paciente había utilizado intermitentemente medias compresivas, pero desde la aparición de su demencia, fue imposible convencerla de ponérselas de nuevo.

El diagnóstico de la úlcera fue fundamentalmente clínico, pues no se contaba con la posibilidad de estudios de ultrasonido Doppler, y se basó en los siguientes criterios diagnósticos: presencia de enfermedad venosa primaria (varicosidades por encima de la lesión hasta la rodilla y lipodermatoesclerosis con el signo de botella de champaña invertida); localización, dimensiones y aspecto de la úlcera (sobre las prominencias óseas, irregular y superficial, con granulación y fibrina en la base); tiempo y forma de evolución; presencia de pulsos periféricos, y ausencia de antecedentes de diabetes o traumatismos.

Antes de la aplicación de la membrana, no se administró ningún tipo de antibiótico de forma tópica ni sistémica y, solo después de aplicada la membrana, se le administró tratamiento antibiótico: una tableta de levofloxacino de 500 mg cada 12 horas por vía oral durante 10 días.

## Procuramiento y preparación del injerto

La membrana amniocoriónica se obtuvo de una donante sometida a una cesárea electiva. Horas antes del inicio de la intervención quirúrgica, a ella se le ofreció información amplia y detallada del uso que se le daría a la membrana placentaria, y su aceptación de la donación quedó expresada en la firma de un documento de consentimiento informado.

En el quirófano se extrajo la placenta bajo condiciones estériles y se depositó en una mesa auxiliar sobre un paño estéril para, posteriormente, diseccionarla separándola de la placenta (figura 2)

Durante la inducción anestésica, se obtuvieron muestras para las pruebas serológicas de HIV-1, HIV-2, HCV, HTLV, sífilis (VDRL y TPHA) y HBV (anticuerpos anti-HBs y anti-HBc). No se consideraron válidos los exámenes serológicos controlados en el último trimestre de embarazo por el riesgo de seroconversion al final del embarazo.

La membrana se lavó tres veces con solución salina al 0,9 % para eliminar restos de sangre. Posteriormente, se dividió en dos fragmentos que se introdujeron en un recipiente estéril que contenía 500 mi de solución salina al 0,9 % y 48 mg de cotrimoxazol, 50 mg de tobramicina y 50 mg de vancomicina, según el método empleado por Rodríguez, *et al*. ^10^. La membrana amniocoriónica permaneció 24 horas a una temperatura de 4 °C en dicha solución.

Según las normas técnicas generales del Ministerio de Salud de Chile para la consecución, preservación e implante de tejidos ^11^, la membrana amniocoriónica obtenida puede mantenerse refrigerada de 36 a 48 horas como máximo después de su obtención, bajo refrigeración entre 2 y 8 °C.

En el momento previo a la implantación de la membrana, esta se lavó tres veces con solución salina al 0,9 % para eliminar restos de la solución con antibióticos en que se preservaba. Se descartaron los fragmentos o partes de estos que contuvieran sangre infiltrada, desgarros u otras alteraciones.

## Implantación de la membrana amniocoriónica

**Figura 3:**
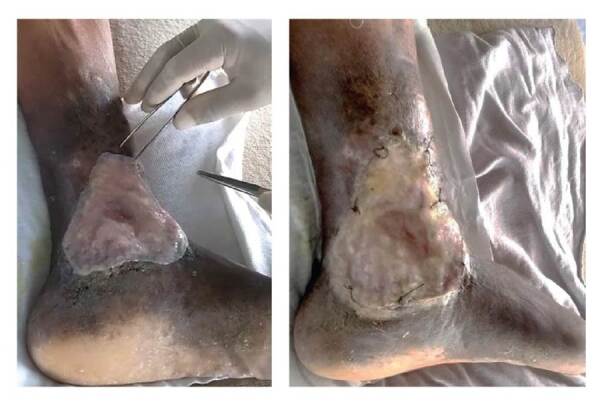
Aplicación de membrana Implantación de la membrana amniocoriónica

Se hizo la limpieza de la úlcera mediante enjuague con solución salina fisiológica estéril, y se procedió al desbridamiento con bisturí de los tejidos necróticos, seguido de un lavado con una solución suave de yodo-povidona (figura 3).

La membrana estéril se implantó asépticamente en el lecho de la úlcera para cubrir toda la superficie de la lesión y el exceso se cortó a la medida con tijeras. Se tuvo el cuidado de evitar la presencia de burbujas de aire debajo de la membrana y esta se aseguró mediante seis puntos de sutura de nailon 3.0 en sus bordes.

La lesión se dejó al descubierto durante unos minutos y luego se cubrió con una gasa con vaselina y una capa de vendaje. La gasa se cambió cada 24 horas hasta el quinto día y, luego, la herida se dejó al descubierto. Tanto la Implantación de la membrana como el seguimiento de la paciente fueron ambulatorios y domiciliarios. Se requirió un único injerto de membrana amniocoriónica.

## Valoración de la cicatrización

Se hizo seguimiento a diario para determinar el tamaño de la úlcera y la tasa de curación. La superficie de la úlcera (largo, ancho y profundidad) se midió en centímetros con una regla graduada, y su área se calculó multiplicando el ancho por el largo. Se definió como curación parcial la reducción del área de la úlcera en un 50 % o menos y, como curación completa, la cicatrización de toda la superficie cruenta.

## Evolución del injerto

**Figura 4:**
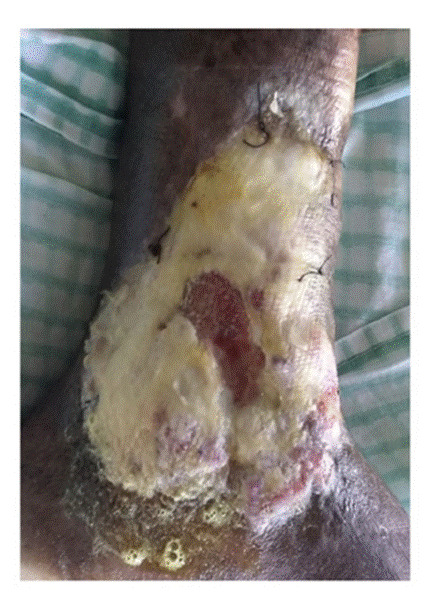
Reabsorción de la membrana

**Figura 5:**
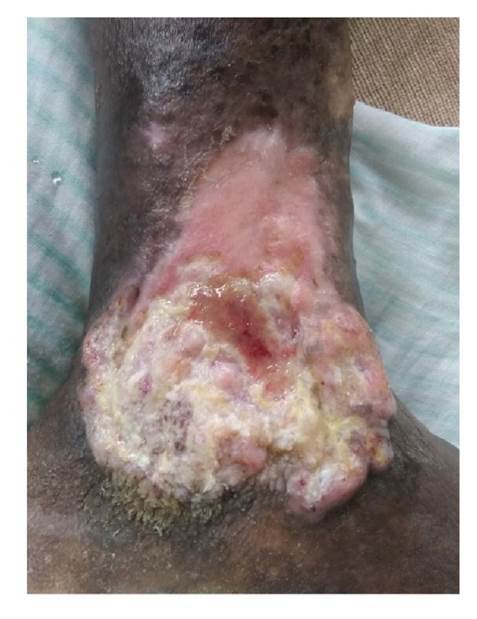
Proceso de cicatrización en el décimo día

**Figura 6:**
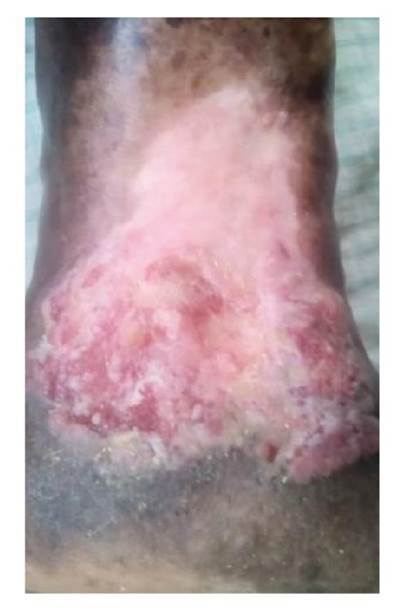
Día 30 de evolución: cicatrización del 50 % de la úlcera

Tras la aplicación del aloinjerto, no hubo signos clínicos locales ni sistémicos de infección o eventos adversos y la lesión evolucionó de la siguiente forma. En el tercer día, la membrana amniocoriónica comenzó a reabsorberse y había desaparecido completamente en el área de mayor profundidad (centro) (figura 4). Hacia el quinto día, se observó reabsorción de la membrana amniocoriónica en los bordes de la úlcera y, en el séptimo, esta se había reabsorbido casi en su totalidad. En el décimo día de evolución, la membrana se había absorbido completamente y la lesión había comenzado a cicatrizar a partir de los bordes, sobre todo en la parte superior. Además, se había formado un exuberante tejido de granulación (figura 5). Hacia el día 30, el proceso de cicatrización había alcanzado el 50 % de la lesión. El centro todavía profundo que se veía en la imagen del décimo día de evolución, se encontraba sellado por un tejido rosado fibroso brillante. En las áreas no cicatrizadas, se observaba un tejido de granulación de buena calidad, sin secreción ni signos clínicos de infección (figura 6).

**Figura 7:**
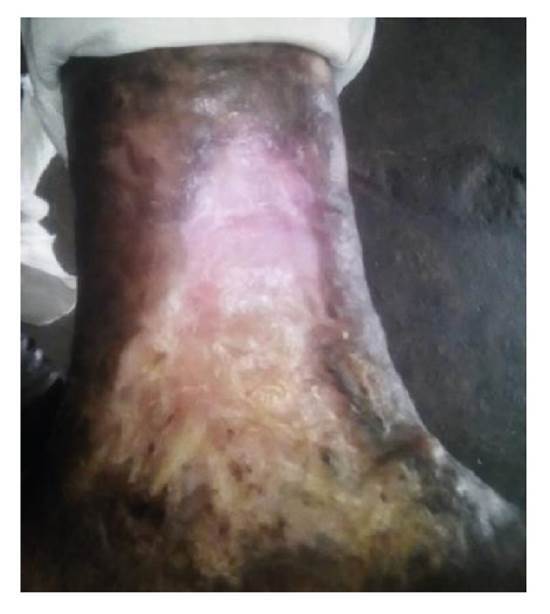
Cicatrización del 100 % de toda la superficie de la úlcera

Durante todo el proceso de cicatrización, se hizo la valoración clínica diaria y se observaron discretas modificaciones en el tejido, hasta que, en el día 60 después de la implantación del aloinjerto de membrana amniótica humana fresca, la úlcera estaba cicatrizada en toda su superficie y se había formado una cicatriz rosada clara en cuyos bordes se apreciaron intentos de pigmentación (figura 7).

## Consideraciones éticas

La paciente y sus familiares otorgaron la autorización para comunicar y difundir la experiencia y los resultados obtenidos con la aplicación del aloinjerto de membrana amniocoriónica en una úlcera venosa crónica de la pierna. El permiso para el uso de los datos clínicos de la paciente, así como de las imágenes de la evolución de la lesión, se obtuvo mediante un documento de consentimiento informado.

## Discusión

En su estado fresco, las membranas amniocoriónicas contienen varias células multipotenciales, factores de crecimiento y proteínas de la matriz extracelular que contribuyen a la cicatrización, además de niveles crecientes de inhibidores de metaloproteinasas de matriz que, en el entorno de la herida, estimulan la proliferación y la migración de células promotoras de la angiogénesis y tienen la capacidad de suprimir reacciones inflamatorias irregulares o no controladas ^12^; es decir, tienen propiedades antiinflamatorias y antifibróticas ^13^. El valor de estas características se ve aumentado por el hecho de que las membranas placentarias también son antimicrobianas y no son inmunogénicas ^14^, lo cual permite su uso como injerto alogénico.

Dada esta evidencia y el alto costo de los tratamientos estándar que no producen la curación completa de la lesión, las membranas amniocoriónicas representan una alternativa versátil, efectiva, asequible, duradera y rápida para el cierre de las úlceras venosas crónicas. Se ha hecho un número considerable de pruebas clínicas para evaluar la seguridad y la eficacia de estas membranas en el contexto de heridas agudas ^15^, úlceras de pie diabético ^16^ y úlceras venosas en las piernas ^17^. Además, los estudios demuestran una mayor tasa de cicatrización de las lesiones en pacientes tratados con membranas amniocoriónicas que con la terapia estándar y los sustitutos de piel ^18^^,^^19^.

En el caso que aquí se informa, la úlcera de miembro inferior tenía 40 años de evolución y había una considerable pérdida de tejido, el cual se había vuelto senescente en el proceso de inflamación o proliferación, perdiendo la capacidad de epitelización después de múltiples medidas terapéuticas, algunas indicadas por el personal médico y otras recogidas de las prácticas populares para la curación de estas lesiones.

Luego de la aplicación de la membrana amniocoriónica, el cierre completo de la úlcera se produjo en un lapso de 60 días. Este resultado es similar al de otros reportes hallados en la literatura. Por ejemplo, en un estudio piloto prospectivo, Mermet*, et al*. ^20^, se evaluaron la seguridad, la viabilidad y los efectos sobre la curación del injerto de membrana amniocoriónica en 15 pacientes con úlceras venosas crónicas en las piernas. El porcentaje de tejido de granulación aumentó significativamente (de 17 % en el día 0 a 69 % en el día 14; *p<*0,0001), y hubo una disminución significativa de la descamación fibrinosa (del 36 % en el día 0 al 16 % en el día 14; *p<*0,001), así como del tamaño de la úlcera y la intensidad del dolor.

Por su parte, Alsina, *et al*. ^21^, hicieron aloinjertos de membrana amniótica en cuatro úlceras vasculares resistentes al tratamiento. En una de las úlceras, la reepitelización completa de la herida se logró en la semana 8; en los otros tres casos, hubo una reducción del 50 % del tamaño en comparación con el valor inicial. En la semana 16, la reducción media en el tamaño de la herida en las cuatro úlceras fue del 81,93 %. La reducción correspondiente en la intensidad del dolor fue del 86,6 %.

El Heneidy, *et al*. ^22^, desarrollaron un estudio controlado, en el cual ninguna de las úlceras del grupo de control mostró reducción en su tamaño. En el grupo experimental, hubo curación completa de 14 úlceras en 14 a 60 días, con una media de 33,3 ± 14,7.

En el estudio de Barbosa, *et al*. ^23^, se evaluó la eficacia del implante de membrana amniótica humana en el proceso inflamatorio, la proliferación de fibroblastos, la formación de colágeno y la reducción de las áreas de heridas de la piel en ratas. El análisis histológico reveló una reducción significativa (p<0,05) en el infiltrado inflamatorio en el grupo experimental en todos los períodos de estudio, en comparación con el grupo de control. Además, en el grupo experimental se presentó un aumento significativo de la proliferación de fibroblastos y del reemplazo de colágeno de tipo III por colágeno de tipo I.

La utilidad de las membranas amniocoriónicas deshidratadas en el tratamiento de las úlceras venosas crónicas, fue evaluada por Serena, *et al*. ^24^, en el 2014. Se trató de un estudio clínico aleatorizado y controlado en 84 pacientes, en el cual se comparó este tipo de aloinjerto aunado al tratamiento estándar con el tratamiento estándar solo. En la semana 4, el 62 % de los pacientes en el grupo con aloinjerto de membranas deshidratadas más tratamiento estándar y el 32 % en el grupo con tratamiento estándar solo, mostraron más del 40 % de cierre de la herida (p=0,005). Además, el grupo con membranas amniocoriónicas y aloinjerto más tratamiento estándar tuvo una reducción de tamaño promedio de 48,1 %, en comparación con el 19 % en el grupo de tratamiento estándar solo. Las úlceras venosas de la pierna tratadas con aloinjerto tuvieron una mejora significativa en la cicatrización a las cuatro semanas, en comparación con la terapia de compresión multicapa sola.

En un estudio posterior, Serena, *et al*. ^25^, evaluaron la correlación correcta entre una tasa intermedia de reducción de la herida (reducción del 40 % del área de la herida después de cuatro semanas de tratamiento) y la curación completa a las 24 semanas, en pacientes con úlcera venosa crónica de la pierna. De 44 pacientes, 20 (45,4 %) presentaron un tamaño de herida reducido mayor o igual al 40 % y, 24 (55 %), una reducción menor del 40 % durante el estudio inicial. La curación completa ocurrió en 16 de 20 (80 %) pacientes del grupo con reducción mayor o igual al 40 %, con una media de 46 días (p=0,0027) y, en 8 de 24 (33,3 %) del grupo con menos de 40 % de reducción, con una media de 103,6 días (p=0,0023).

En el presente estudio, se empleó membrana amniocoriónica fresca; sin embargo, ha habido experiencias de resultados variables con membranas procesadas (criopreservación, almacenamiento hipotérmico y deshidratación, o acelular). Es por ello que tanto el procesamiento como la composición de los injertos amnióticos han suscitado un importante debate. Se presume que el procesamiento altera la composición proteómica y, en consecuencia, puede afectar el funcionamiento de los injertos ^26^. Según McQuilling, *et al*. ^27^, la mayoría de los productos de membranas placentarias disponibles en el mercado son obtenidos por deshidratación o liofilización, lo que altera las características en diversos grados; las capas incluidas en estos injertos varían y la mayoría contiene un amnios, o amnios y coriones de doble capa, o la capa de amnios sola.

En los estudios de Lim, *et al*. ^28^, Russo, *et al*. ^29^, y Paolin, *et al*. ^30^, se ha comparado el amnios deshidratado con el amnios fresco o el criopreservado, y se encontraron cambios significativos en la estructura de las membranas, así como una disminución en el contenido de citocinas después de la deshidratación. Según Alien, *et al*. ^31^, después de la criopreservación, las membranas presentan el 77 % de disminución en las proteínas, en comparación con las membranas frescas.

En cuanto a la seguridad del injerto de membrana amniocoriónica fresca, se ha podido concluir que es un procedimiento seguro, como lo constataron Mermet, *et al*. ^20^, y Alsina, *et al*. ^21^, al reportar la ausencia de efectos adversos con la implantación de la membrana fresca.

Según Therani, *et al*. ^32^, aunque la membrana amniótica intacta siempre ha sido una opción adecuada para muchos estudios clínicos y experimentales, las modificaciones adicionales permiten ampliar sus aplicaciones. Sin embargo, los altos costos de estos productos comerciales limitan el acceso a esta terapia, por lo que la aplicación de membranas placentarias frescas resulta una indiscutible alternativa, ya que, con métodos de procesamiento de bajo costo, se puede garantizar la seguridad biológica adecuada (ausencia de agentes microbiológicos) para su uso en humanos.

Por tal motivo, es necesario aportar evidencia sobre alternativas a los productos comerciales y procurar la disponibilidad de membranas placentarias para los pacientes de bajos recursos, con el fin de ofrecerles la oportunidad de curación y la disminución de los problemas de discapacidad que ocasionan las úlceras crónicas de los miembros inferiores. En el caso que se describe, se demostró la acción efectiva de la membrana amniocoriónica en una úlcera intratable de 40 años de evolución.

## Conclusión

El aloinjerto de membrana amniocoriónica humana fresca es una alternativa terapéutica para la curación de las úlceras vasculares crónicas persistentes de extremidades inferiores. Su implantación no se asocia con efectos adversos y se puede obtener a un menor costo que las membranas comerciales procesadas.
